# Psychiatric symptoms and intentions to quit smoking: How regularity and volume of cigarette consumption moderate the relationship

**DOI:** 10.18332/tid/163258

**Published:** 2023-06-14

**Authors:** Xiaochen Yang, Lanchao Zhang, Hao Lin, Haoxiang Lin, Wangnan Cao, Chun Chang

**Affiliations:** 1 Department of Social Medicine and Health Education, School of Public Health, Peking University, Beijing, China; 2 Institute for Global Health and Development, Peking University, Beijing, China

**Keywords:** intention to quit, smoking cessation, moderating effect, depression, anxiety

## Abstract

**INTRODUCTION:**

Smoking prevalence is disproportionately higher in those with psychiatric symptoms. Smokers with psychiatric symptoms are less likely to have an intention to quit smoking and attain eventual smoking abstinence. This study presents the relationship between depressive/anxiety symptoms and the intention to quit smoking and related influencing factors.

**METHODS:**

A cross-sectional study recruited 931 current smoking individuals covering two provinces in China in July 2022. The online survey comprised questions concerning sociodemographic characteristics, smoking conditions and psychiatric symptoms. Chi-squared analyses and moderation analyses were applied.

**RESULTS:**

The proportion of smokers who had the intention to quit smoking within six months was 46.1%. In comparison with subjects without depressive/anxiety symptoms, individuals who had both psychiatric symptoms were less likely to have the intention to quit smoking (39.3% vs 49.8%, χ^2^=9.130, p=0.028). As for the moderating model of depression, the interaction term of depressive symptoms and smoking regularly was significant (*β*=0.554, t=3.260, p=0.001). For those who were occasional smokers, depressive symptoms significantly lowered their quitting intentions. The regularity of smoking similarly moderated the effect of anxiety symptoms on quitting intentions. When the number of cigarettes used weekly served as the moderator, the interaction of this number and depressive symptoms was significant, as well as for anxiety (p<0.001), indicating that the volume of cigarette consumption moderated the relationship between depressive/anxiety symptoms and intention to quit smoking.

**CONCLUSIONS:**

Psychiatric symptoms were significant factors in reducing the willingness of smokers to quit smoking, and the effects were moderated by smokers’ cigarette consumption conditions. Interventions are urged to enhance quitting intentions of these vulnerable smokers.

## INTRODUCTION

Tobacco use remains a public health problem prevalent worldwide, entailing severe health risks and heavy social burdens^1^. Smoking cigarettes contributes significantly to the morbidity and mortality rates of non-communicable diseases^2^. As the only way to prevent harm to health, smoking cessation and treatment for co-morbidities are key priorities.

Previous research suggests that, on average, a smoker needs ≥30 quitting attempts before successfully achieving abstinence for at least one year^3^. It has been shown that smoking cessation is a difficult and complex process, and smokers generally apply many methods and approaches to achieving cessation. And quitting intention presents to be essential for smoking cessation^4^.

Initial quitting intentions are challenging to rise due to complicated impacting factors, including individual demographic characteristics, smoking conditions, and social and cultural norms^5-7^. Smoking-related conditions have also proved to affect quitting intentions, such as the heaviness and duration of smoking. Previous studies have indicated that the intention to quit smoking was lower at higher levels of smoking heaviness^8,9^ and nicotine dependence^7,10^.

Consistent findings have demonstrated that those with mental health disorders are more likely to be smokers^2,11,12^. Smoking rates among those with mental health disorders were more than twice the rates of those without mental health disorders^13^. As a susceptible population, psychiatric smokers were less likely to intend to quit smoking, and this population’s smoking rate declined much more slowly than the general population^14^. People who experienced psychiatric symptoms were more likely to try smoking to alleviate stress or frustration, which could, in turn, lead to a lower willingness to quit^15^.

Some researchers have suggested that psychiatric smokers are also motivated to quit but lack the availability of appropriate treatment for quitting^12,16^, especially among stress-precipitated beginners. Smokers with psychiatric symptoms may derive comfort from tobacco use to release withdrawal symptoms and may form a habit of smoking to seek composure. Advances in neuroscience suggest that dopamine release, stimulated by substances such as nicotine, gradually diminishes, reducing sensitivity and leading to increased cigarette consumption^17,18^. A study targeted at smokers with mental illnesses showed that lower breath CO was a significant predictor of quit attempts, indicating that psychiatric patients who had a relatively lighter smoking intensity were more likely to try to quit^19^. The evidence above provided clues that smoking heaviness might play the role of moderator in the relationship between psychiatric symptoms and quitting intentions. An exploratory test is needed to shed light on the relationship between psychiatric symptoms and the intention to quit.

This study aimed to explain the underlying interaction effects of smoking conditions and psychiatric symptoms on quitting intentions. First, we assessed how well the sociodemographic characteristics and smoking conditions are associated with smokers’ quitting intentions. Second, we explored the distribution disparities of quitting intentions among smokers with/without depressive/anxiety symptoms. Then we tested the relationships between psychiatric symptoms and quitting intentions among smokers, moderated by smokers’ tobacco consumption regularity and volume.

## METHODS

### Target population and sample size

This study was conducted as a pre-experiment of a large-scale randomized controlled trial, and the convenient sampling method was used. Current smokers were recruited in Jiangsu and Shandong provinces in eastern China in July 2022. The inclusion criteria were: 1) age ≥18 years; and 2) current smokers having used at least one cigarette in the past week.

### Measures

A self-administered questionnaire including sociodemographic characteristics, the intensity of cigarette consumption, smoking quitting intentions, depression and anxiety was designed by the research team. Quitting intentions were assessed using the question: ‘How likely are you to quit smoking in the next six months?’^9^, with five response items that ranged from ‘completely not likely’ to ‘very likely’. Depressive symptoms were measured by the brief 10-item version of the Center for Epidemiologic Studies Depression Scale (CES-D-10). An example item is: ‘I was bothered by things that usually don’t bother me’. Participants were asked to answer how often (days) they have felt this way during the past week on a scale from 0 (<1d) to 3 (5–7 d), with items 5 and 8 reverse coded. We used the cutoff point of 10 to qualify for having depressive symptoms^20^. Anxiety symptoms were assessed by the scale of Generalized Anxiety Disorder (GAD-7) with a score from 0 (totally not) to 3 (almost every day) for 7 items^21^. The total score was calculated, and three cutoff points, 5, 10, and 15, represented mild, moderate, and severe levels of anxiety. In this study, we focused on anxiety symptoms rather than illness, and considering that a score of ≥5 was the criterion for determining the presence of anxiety symptoms, we used a score of 5 as the cutoff point^22^. Both CES-D-10 and GAD-7 have been widely used and shown satisfactory validity and reliability in the Chinese population ^23,24^.

To investigate the intensity of cigarette consumption, we considered two aspects of smoking behavior: regularity and volume of smoking. Smoking regularity was assessed using the question: ‘Do you smoke regularly? (yes/no); which means you smoke a fixed number of cigarettes each day’^25^. Participants were also asked about the number of cigarettes they consumed per day (for regular smokers) and per week (for occasional smokers).

Sociodemographic characteristics included age, sex (male, female), education level (high school or lower, Bachelor’s, Master’s or higher), marital status (single/divorced/widowed, married) and monthly income level (<3000, 3000–5999, 6000–8999, and >9000 RMB; 1000 Chinese Renminbi about US$140).

Family members’ smoking conditions were measured by the question: ‘How many family members who are current smokers do you live with?’.

### Data collection

This study was based on a self-administered questionnaire. We deployed this survey on the online survey platform ‘Wenjuanxing’. A total of 931 smokers aged >18 years completed the questionnaire with convenience sampling. After data cleaning, 924 (99.2%) valid questionnaires were included in the analysis. Seven questionnaires were excluded due to missing data.

### Statistical analysis

Chi-squared analyses were used to identify differences in sociodemographic characteristics and the variables of smoking behaviors among those with/without the intention to quit smoking. A dichotomous variable for having quitting intentions (the last two response categories) versus no intentions (the first three categories) was created based on the question mentioned in the measures section. Low cigarette consumption level was categorized as ≤25th percentile of the number of cigarettes smoked per week, high level ≥75th percentile and medium 25th< percentile <75th. The psychiatric disparities of the participants who had/did not have the intention to quit smoking were also analyzed. All included participants completed the full measuring items of CES-D-10. Participants were separated into two categories: 1) smokers with depressive symptoms (total score ≥10), and 2) smokers without depressive symptoms (total score <10). Participants with/without anxiety symptoms were categorized in a similar way based on the scale of GAD-7. The statistical analyses above were performed using IBM SPSS 26.0.

The moderating effects of regularity and volume of cigarette consumption on the relationship between depressive/anxiety symptoms and quitting intentions were tested using Mplus 8.3. Smoking volume was measured as the total number of cigarettes consumed per week by participants and served as a continuous variable in the moderating models. First, regression models were tested to examine the association between depressive/anxiety symptoms and quitting intentions. Covariate variables included age, sex, education level, marital status, monthly income level, and number of smokers in the family. Then, each moderator (smoking regularity, number of cigarettes used per week) and corresponding interaction items were added to the models mentioned above to identify moderating effects on quitting intentions. When moderating effects were significant, simple slope tests were used. Analyses were performed with Mplus using the robust maximum likelihood (MLR) method, which was proven accurate in models with mixed-item response types^26^. Differences were considered statistically significant for two-tailed tests with p<0.05.

## RESULTS

Of the 924 participants, the prevalence of depression and anxiety was 34.2% and 41.5%, respectively, and 426 (46.10%) smokers intended to quit smoking within six months. Smokers who were female, younger, had a Bachelor’s degree or higher and were not married, were more likely to have quitting intentions. Participants who had quitting intentions were comparable to those who did not have quitting intentions in terms of income and absence/presence of smoking family members. The rate of having quitting intentions was lower in regular smokers compared with participants who occasionally smoked (26.17 % vs 59.00 %, χ^2^= 95.603, p<0.001). Smokers with high cigarette consumption were significantly less likely to intend to quit smoking (23.55 % vs moderate 47.92% vs low 64.41%, χ^2^=91.427, p<0.001). The general characteristics and comparisons of quitting intentions are presented in Table 1.

**Table 1:** Sociodemographic characteristics and quitting intentions of participants based on a sample of adult smokers in China, 2022 (N=924)

*Characteristics*	*All n (%)*	*Having quitting intentions in the next 6 months*		*χ^2^*
		*No n (%)*	*Yes n (%)*	
**Total**	924 (100)	498 (53.90)	426 (46.10)	
**Sex**				19.600
Male	704 (76.20)	408 (57.95)	296 (42.05)	
Female	220 (23.80)	90 (40.91)	130 (59.09)	
**Age** (years)				20.473
18–24	74 (8.00)	36 (48.65)	38 (51.35)	
25–44	621 (67.20)	309 (49.76)	312 (50.24)	
≥45	229 (24.80)	153 (66.81)	76 (33.19)	
**Education level**				14.284
High school or lower	195 (21.10)	128 (65.64)	67 (34.36)	
Bachelor’s	639 (69.20)	321 (50.23)	318 (49.77)	
Master’s or higher	90 (9.70)	49 (54.44)	41 (45.56)	
**Marital status**				9.090
Single/divorced/widowed	204 (22.10)	91 (44.61)	113 (55.39)	
Married	720 (77.90)	407 (56.53)	313 (43.47)	
**Monthly income level** (RMB)				0.297
<3000	102 (11.00)	56 (54.90)	46 (45.10)	
3000–5999	297 (32.10)	158 (53.20)	139 (46.80)	
6000–8999	269 (29.10)	148 (55.02)	121 (44.98)	
>9000	256 (27.70)	136 (53.13)	120 (46.88)	
**Smoke regularly**				95.603
No	561 (60.70)	230 (41.00)	331 (59.00)	
Yes	363 (39.30)	268 (73.83)	95 (26.17)	
**Cigarette consumption level**				91.427
Low	281 (30.40)	100 (35.59)	181 (64.41)	
Medium	384 (41.60)	200 (52.08)	184 (47.92)	
High	259 (28.00)	198 (76.45)	61 (23.55)	
**With at least one smoking family member**				0.266
No	523 (56.60)	278 (53.15)	245 (46.85)	
Yes	401 (43.40)	220 (54.86)	181 (45.14)	

RMB: 1000 Chinese Renminbi about US$140.

**Figure 1:**
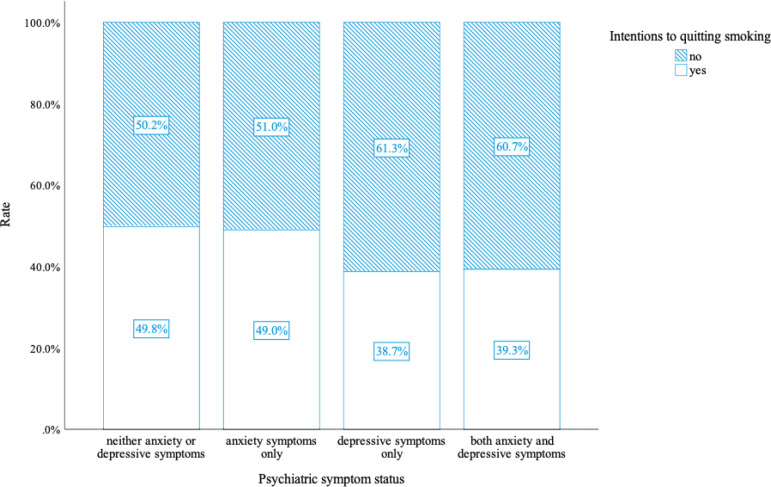
Quitting intentions of smokers by different mental health status, China, 2022 (N=924)

As shown in Figure 1, compared to smokers without depressive or anxiety symptoms, those with both depressive and anxiety symptoms were less likely to have a quitting intention (39.3% vs 49.8%, p<0.05). Smokers with only depressive symptoms showed the lowest rate of intention to quit smoking (38.7%) compared with those of other psychiatric symptoms (p<0.05).

**Figure 2:**
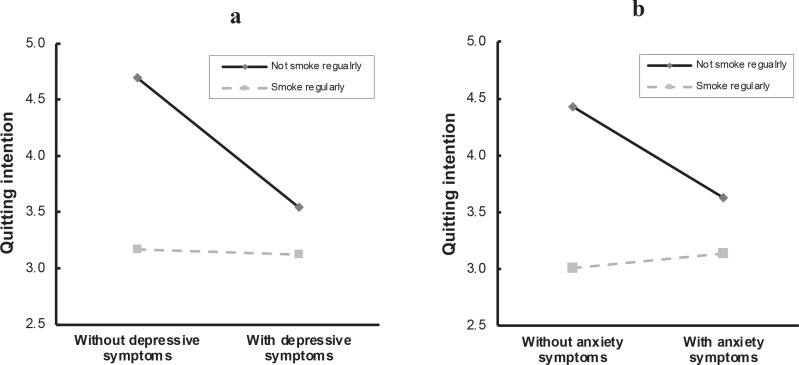
The interaction effect between smoking regularity and depressive/anxiety symptoms on quitting intentions, China, 2022 (N=924)

Sociodemographic factors and family members’ smoking status were adjusted as covariates in the moderating models. Depressive symptoms had a negative effect on quitting intentions (*β*= -0.578, t= -4.776, p<0.001), and the interaction term of depressive symptoms and smoking regularity was significant statistically (*β*=0.554, t=3.260, p=0.001) (Table 2). Simple slope tests indicated that depressive symptoms apparently lowered occasional smokers’ quitting intentions, and the slope difference between regular and occasional smokers was significant statistically (t=3.260, p=0.001) (Figure 2a). Similarly, the regularity of smoking moderated the effect of anxiety symptoms on quitting intentions (*β*=0.464, t=2.822, p=0.005) (Table 2). Figure 2b shows the results of simple slope tests using the pick-a-point approach.

**Table 2:** Moderating effect of smoking regularity on the relationship between depressive/anxiety symptoms and quitting intentions, China, 2022 (N=924)^a^

	*Depression*				*Anxiety*			
	*β*	*SE*	*t*	*p*	*β*	*SE*	*t*	*p*
**Predictor**								
Psychiatric symptoms (PS)	-0.578	0.121	-4.776	<0.001	-0.397	0.117	-3.389	0.001
Regularity	-0.973	0.111	-8.745	<0.001	-0.955	0.115	-8.297	<0.001
PS × Regularity	0.554	0.170	3.260	0.001	0.464	0.165	2.822	0.005
**Simple slope tests**								
Smoke occasionally	-0.578	0.121	-4.776	<0.001	-0.397	0.117	-3.389	0.001
Smoke regularly	-0.024	0.122	-0.197	0.844	0.068	0.117	0.579	0.563
Difference	0.554	0.170	3.260	0.001	0.464	0.165	2.822	0.005

a Model adjusted for age, sex, education level, marital status, income level and number of smoking families.

**Figure 3:**
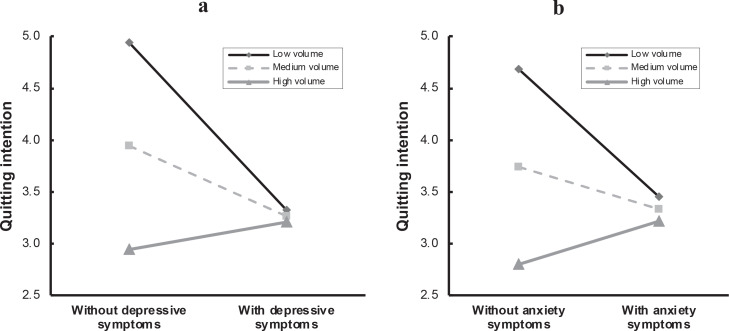
The interaction effect between smoking volume and depressive/anxiety symptoms on quitting intentions, China, 2022 (N=924)

When the number of cigarettes consumed weekly served as the moderator, there was a statistically significant interaction effect between this number and depressive symptoms (*β*=0.008, t=5.080, p<0.001) (Table 3). Given that the moderator was a continuous variable, the three points selected in the simple slope tests were the mean-SD, mean, and mean+SD, corresponding to low, medium and high levels of cigarette consumption (Figure 3a). Simple slope tests showed that depressive symptoms significantly reduced quitting intentions among smokers of low and medium cigarette consumption compared with smokers of high consumption (t=5.080, p<0.001).

**Table 3:** Moderating effect of smoking volume on the relationship between depressive/anxiety symptoms and quitting intentions, China, 2022 (N=924)^a^

	*Depression*				*Anxiety*			
	*β*	*SE*	*t*	*p*	*β*	*SE*	*t*	*p*
**Predictor**								
Psychiatric symptoms (PS)	-0.338	0.088	-3.849	<0.001	-0.203	0.084	-2.409	0.016
Volume	-0.009	0.001	-8.067	<0.001	-0.009	0.001	-7.576	<0.001
PS × Volume	0.008	0.002	5.080	<0.001	0.007	0.002	4.169	<0.001
**Simple slope tests**								
Low volume	-0.786	0.135	-5.828	<0.001	-0.601	0.136	-4.413	<0.001
Medium volume	-0.338	0.088	-3.849	<0.001	-0.203	0.084	-2.409	0.016
High volume	0.110	0.113	0.977	0.328	0.194	0.118	1.648	0.099
Difference	0.896	0.176	5.080	<0.001	0.795	0.191	4.169	<0.001

a Model adjusted for age, sex, education level, marital status, income level and number of smoking families.

The interaction effect was similar in cigarette consumption volume and anxiety symptoms (t=4.169, p<0.001), indicating that cigarette consumption volume moderated the relationship between anxiety symptoms and intention to quit smoking (Table 3). Figure 3b shows that anxious participants were greatly less likely to intend to quit smoking than non-anxious smokers when they were at the same relatively low level of cigarette consumption.

## DISCUSSION

Among the 924 smokers who participated, regular smokers accounted for 39.3% and less than half of the participants had intentions to quit smoking within six months (46.10%). Our results suggest that the prevalence of depression and anxiety among participants was much higher than the figures reported in nationally representative epidemiological data^27^. Specifically, depression was reported by 34.2% of participants, compared with 3.6% in national data, while anxiety was reported by 41.5% of participants, compared with 5.0% in national data. These findings are consistent with previous research suggesting that individuals with mental health disorders are more likely to smoke^12^.

Our study suggests that smokers with only depressive symptoms report the lowest rate of intention to quit smoking (38.7%), while smokers with both depressive and anxiety symptoms report the second lowest rate (39.3%). Previous studies have shown that anxiety, stress, and depression contribute highly to initial cigarette consumption neurochemically^28,29^. In addition, smokers with psychiatric symptoms have greater difficulty quitting smoking. As we all know, quitting intentions play a key role in the smoking cessation process^30^. Previous studies have shown that intention to quit strongly predicts a quit attempt and further conversion to eventual abstinence ^31,32^. Quit attempts preceded by an intention to quit smoking are much more likely to achieve abstinence compared with a sudden or unplanned quit attempt^33^. Previous studies have reported that choosing ‘yes’ to the question measuring quitting intentions might indicate a commitment to quitting^33,34^. Unfortunately, psychiatric smokers find it difficult to take on a commitment to quitting smoking and maintain the habit. They indulge in cigarettes, have no motivation to quit smoking, and continue chronic tobacco use^29^.

This study demonstrates that being female, younger, more educated, and not married significantly predicted having quitting intentions (p<0.05). Our results are comparable with earlier studies^8,9^, regardless of the inconsistency of existing literature with regard to sociodemographic factors across countries^35^. Although income levels and living with/without smokers did not present significant associations with quitting intentions, they were included in further moderating effect analyses as covariables, having been proved to be correlated with intentions in other studies^36^. Regarding tobacco use, we found that smokers with a high level of cigarette consumption and regular daily smoking patterns were significantly less likely to intend to quit smoking. Our findings are consistent with other studies^8,9^, indicating that the likelihood of intending to quit is greater if a smoker consumes fewer cigarettes and smokes occasionally.

Our results suggest that psychiatric symptoms do not independently influence quitting intentions; those with different levels of smoking intensity reported varying willingness to quit when taking into account the influence of psychiatric symptoms. Smokers with psychiatric symptoms had lower quitting intentions, and relatively low intensity of smoking exhibited significant moderating effects. From the psychoanalysis point of view, substance use is an escape from intolerable internal factors and a defence against psychological suffering for psychiatric patients^37^. The theory of self-medication considers psychiatric patients using substances to relieve distressing symptoms, which is an artificial defence against overwhelming effects, such as intense rage, embarrassment, or hurt and rejection^38^. This pattern provides insight into understanding psychiatric smokers’ tobacco use behaviors. Relieving psychiatric symptoms may take priority over attempting to quit smoking for some smokers with depressive or anxiety symptoms. Individuals with mental health conditions tend to be driven by compulsive cigarette use, extract more nicotine per puff, and become nicotine dependent more easily^39^.The instinct to dampen depression and anxiety, seek comfort and escape anticipatory fear of withdrawal symptoms work together, inducing repeated and habitual cigarette use behaviors. We could infer that cigarette smoking has the capacity to alleviate psychological symptoms, weakening intentions to quit and gradually becomes addicting, which provides an explanation for the moderating effects of irregular smoking and low smoking volume. Although the self-treatment hypothesis has also been questioned for lack of robust empirical evidence^40^, this theory offers a rational explanation for our findings.

Understanding that smoking intensity could moderate the effects of psychiatric symptoms on quitting intentions may assist with tailored smoking cessation guidance for smokers with psychiatric symptoms. Our findings suggest that preventing the change from an occasional smoker to a heavy and habitual smoker is an important task in treating smokers with psychiatric symptoms or disorders. The explanatory hypothesis from the identified moderating relationship is that those experiencing depressive/anxiety symptoms may feel they need to address these symptoms before seeking treatment for smoking cessation or attempting to quit. From a neuroscientific point of view, anxiety and depressive feelings triggered by repeated smoking can merge with initial psychological disorders, making psychiatric smokers vulnerable to addiction and more difficult to quit smoking^17,18,29^. Our findings highlight a further concern that existing tobacco control strategies rarely include mental health treatment^22^ even though mental health problems appear to be a barrier to smoking cessation. Previous research has revealed that <1% of depression patients in China have received adequate treatment, regardless of medication or psychotherapy^41^. It is urged that the importance of improving mental health be highlighted, as well as increasing the rate of successful smoking cessation. Therefore, appropriate interventions that have been shown to simultaneously address substance use and mental health symptoms are needed to address multiple treatment objectives (e.g. Motivational Interviewing and Acceptance and Commitment Therapy).

Furthermore, while depression and anxiety decrease quitting intentions more greatly when participants are of low-intensity smoking, smokers in such a state still have higher scores of quitting intentions on average. It is a chance, as a higher willingness to quit smoking would induce more active quitting attempts, and it is expected that tailored intervention targeting the populations have the capacity to induce more smoking cessation success. Although there is a complex set of factors that affect the intentions to quit smoking and practical action, our estimates can be considered inspiring and meaningful in terms of implications for understanding specific impact mechanisms and processes for psychiatric smokers. Efforts are required in all aspects, such as comprehensive medical treatment settings, offering both mental health care and smoking cessation aids, or counselling covering these areas, such as Motivational Interviewing and Cognitive Behavioral Therapy, to ultimately reduce smoking prevalence and health inequalities in this population.

### Limitations

This study still has several shortcomings limiting the extrapolation of the findings. First, the survey was cross-sectional and may result in recall or information bias. The self-administered questionnaires were completed through an online platform, which could attract younger participants. Secondly, our samples were not completely representative due to the convenience sampling method. Third, self-reported scales could not make clinical diagnoses of mental health disorders, for which reason we used the term psychiatric symptoms rather than ‘diagnosed’ depression/anxiety. Due to these limitations, the inherent mechanism of how psychiatric disorders affect initiating to quit still requires further longitudinal research to clarify with certainty.

## CONCLUSIONS

The results of our study demonstrate that psychiatric symptoms have a unique decreasing effect on intentions to quit smoking among smokers with different intensities of cigarette consumption. Relatively low regularity and volume of cigarette consumption were observed to significantly interact with depression and anxiety in reducing intentions to quit smoking. It is important for future research to continue to estimate psychiatric smokers’ cigarette consumption status and examine their related quitting motivations. Interventions should be taken to increase the willingness of these vulnerable smokers to quit. Motivation to quit smoking should be stimulated and strengthened among psychiatric smokers so as to improve smoking cessation rates.

## Data Availability

The data supporting this research are available from the authors on reasonable request.
